# Gliding Motility of *Babesia bovis* Merozoites Visualized by Time-Lapse Video Microscopy

**DOI:** 10.1371/journal.pone.0035227

**Published:** 2012-04-10

**Authors:** Masahito Asada, Yasuyuki Goto, Kazuhide Yahata, Naoaki Yokoyama, Satoru Kawai, Noboru Inoue, Osamu Kaneko, Shin-ichiro Kawazu

**Affiliations:** 1 National Research Center for Protozoan Diseases, Obihiro University of Agriculture and Veterinary Medicine, Inada, Obihiro, Japan; 2 Laboratory of Molecular Immunology, Graduate School of Agricultural and Life Sciences, The University of Tokyo, Yayoi, Bunkyo-ku, Tokyo, Japan; 3 Department of Protozoology, Institute of Tropical Medicine (NEKKEN) and the Center of Excellence Program, Nagasaki University, Sakamoto, Nagasaki, Japan; 4 Laboratory of Tropical Medicine and Parasitology, Dokkyo University School of Medicine, Mibu, Shimotsuga, Tochigi, Japan; Weill Cornell Medical College, United States of America

## Abstract

**Background:**

*Babesia bovis* is an apicomplexan intraerythrocytic protozoan parasite that induces babesiosis in cattle after transmission by ticks. During specific stages of the apicomplexan parasite lifecycle, such as the sporozoites of *Plasmodium falciparum* and tachyzoites of *Toxoplasma gondii*, host cells are targeted for invasion using a unique, active process termed “gliding motility”. However, it is not thoroughly understood how the merozoites of *B. bovis* target and invade host red blood cells (RBCs), and gliding motility has so far not been observed in the parasite.

**Methodology/Principal Findings:**

Gliding motility of *B. bovis* merozoites was revealed by time-lapse video microscopy. The recorded images revealed that the process included egress of the merozoites from the infected RBC, gliding motility, and subsequent invasion into new RBCs. The gliding motility of *B. bovis* merozoites was similar to the helical gliding of *Toxoplasma* tachyzoites. The trails left by the merozoites were detected by indirect immunofluorescence assay using antiserum against *B. bovis* merozoite surface antigen 1. Inhibition of gliding motility by actin filament polymerization or depolymerization indicated that the gliding motility was driven by actomyosin dependent process. In addition, we revealed the timing of breakdown of the parasitophorous vacuole. Time-lapse image analysis of membrane-stained bovine RBCs showed formation and breakdown of the parasitophorous vacuole within ten minutes of invasion.

**Conclusions/Significance:**

This is the first report of the gliding motility of *B. bovis.* Since merozoites of *Plasmodium* parasites do not glide on a substrate, the gliding motility of *B. bovis* merozoites is a notable finding.

## Introduction

The apicomplexan phylum contains obligate intracellular parasites that are major pathogens of humans and livestock. During specific stages of the apicomplexan parasite lifecycle, host cells are targeted for invasion using a unique, active process termed “gliding motility”. Gliding motility by apicomplexan parasites does not require shape changes like the crawling of amoebae, nor do the zoite stages of these parasites have cilia or flagella [1]. Instead, the motility is driven by coupling the translocation of surface adhesins to an actomyosin motor beneath the parasite plasma membrane [2]. Gliding motility has been reported in *Plasmodium* spp. (sporozoite and ookinete), *Toxoplasma gondii* (tachyzoite and sporozoite), *Cryptsporidum parvum* (sporozoite), and *Eimeria* spp. (sporozoite) [3], [4], [5], [6].

*Babesia bovis* is an apicomplexan intraerythrocytic protozoan parasite that induces babesiosis in cattle after transmission by ticks. *B. bovis* is a representative of the large-type *Babesia* species. Sporozoites of the *Babesia* parasite directly invade host red blood cells (RBCs), and all parasitic stages in the vertebrate host develop in the RBCs [7]. Although the precise timing is unknown, once merozoites invade a RBC, the parasite rapidly escapes from the parasitophorous vacuole (PV) that is formed by invagination of the RBC membrane during invasion [8]. Following establishment of the free parasite within a host RBC, *B. bovis* produces two merozoites by binary fission. After erythrocytic lysis, each merozoite invades a new RBC and successive merogonies occur [7]. However, the process of merozoite entry into RBCs is not thoroughly understood, and gliding motility has so far not been recorded in *Babesia* parasites.

Gliding motility can be observed by time-lapse video microscopy or easily detected by staining for surface components of the parasites that are deposited in trails left on the substrate [9]. In *T. gondii* tachyzoites, three forms of gliding motility have been characterized by video microscopy analysis: circular gliding, twirling, and helical gliding [4]. Since actin filaments are required for gliding, chemical agents that disrupt actin filament polymerization, such as cytochalasins and latrunculins, can inhibit the motility [10]. In recent years, genetic manipulation methodologies have advanced in apicomplexan parasites and fluorescently labeled parasite populations are being employed in time-lapse imaging analysis. In *Plasmodium*, green fluorescent protein (GFP)-expressing parasites have given unprecedented insight into their behavior within mammalian hosts and vector mosquitoes [11], [12].

Here we have observed gliding motility using time-lapse video microscopy of GFP-expressing *B. bovis* merozoites that were developed in our previous study [13]. Time-lapse video images delineated the sequential process of parasite-infected RBC (IRBC) rupture, merozoite egress from IRBCs, gliding motility of the merozoites, attachment and invasion of merozoites into new RBCs, and formation and breakdown of PVs.

## Results

### *B. bovis* Merozoites Glide to Infect RBC

**Figure 1:**
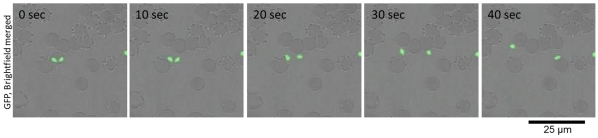
Time-lapse video microscopy of a *B. bovis* merozoite engaged in gliding. *In vitro* cultured IRBCs were placed on a glass slide and their motility documented with confocal laser microscopy. The time elapsed between each frame is indicated in seconds. Gliding of the *B. bovis* merozoites is characterized as forward movement with the apical end at the front position.

**Figure 2:**
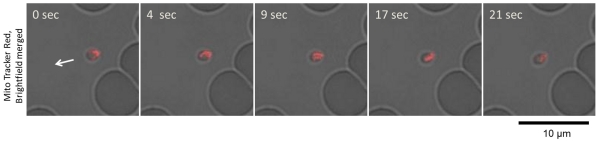
Helical motility of *B. bovis* merozoites. *In vitro* cultured IRBCs were stained with a fluorescent mitochondrial probe (MitoTracker Red) and placed on a glass slide for confocal laser microscopy analysis. The shift in red fluorescence on the merozoites (top to bottom) indicates helical motility of the merozoites. The arrow indicates the direction of merozoite movement.

From observations of the GFP-expressing *B. bovis* populations, we could confirm the gliding motility of the extra-erythrocytic merozoites *in vitro*. To characterize the mode of gliding motility, cultured IRBCs were applied to glass slides and egress of the merozoites from the IRBC and their subsequent motility were recorded by confocal laser microscopy. By analyzing the video recordings, straight or meandering motility, but not retreating movement, was observed (Fig. 1 and Video S1). The apical end of the merozoites was at the front position in the direction of movement. To characterize the gliding mode of *B. bovis* merozoites, parasite mitochondria were stained with MitoTracker Red. The red fluorescence-stained organelles in the cells enabled us to track the body rotation around the long axis. The merozoites rotated in a counterclockwise direction with respect to the direction of travel (Fig. 2 and Video S2). The motility observed in the *B. bovis* merozoites was similar to helical gliding, but other modes of gliding such as circular gliding or twirling were not observed.

### *B. bovis* Merozoites form Trails During Gliding

**Figure 3:**
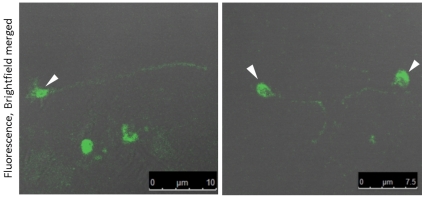
*B. bovis* merozoites deposited surface membrane trails during gliding on a solid substrate. An indirect immunofluorescence antibody assay was performed by using anti-*B. bovis* MSA-1 mouse antiserum. Arrow heads show the fluorescence from the merozoites.

To confirm that the movement of *B. bovis* merozoites is due to gliding, the presence of gliding trails was investigated by an indirect immunofluorescence antibody test using anti-merozoite surface antigen 1 (BbMSA-1) antibody. IRBCs were placed on poly-L-lysine-coated glass slides and the merozoites that egressed from RBCs were allowed to glide on the slides. The slides were stained with the antibody. As a result, *B. bovis* merozoites were observed to deposit trails that formed straight or winding patterns in accordance with observations from time-lapse video microscopy (Fig. 3).

### Gliding Motility of *B. bovis* Merozoites Relies on the Parasite’s Actin Cytoskeleton

The gliding speed of the merozoites was not constant because the parasites paused and moved with variable velocity. However, the maximum net forward speed of the gliding motility was estimated to be 1.2 µm/sec under the assay conditions (Table 1). In order to determine whether gliding motility of *B. bovis* merozoites depended on an actomyosin motor system, the effect of an actin filament depolymerizer (cytochalasin D) and polymerizer (jasplakinolide) on merozoite motility was analyzed. Motility of *B. bovis* merozoites was completely blocked by 1 µM cytochalasin D or 1 µM jasplakinolide, indicating that the motility was driven by an actomyosin motor. The IRBCs incubated with 1 µM of cytochalasin D showed unsuccessful egress of the merozoites and even rupture of the IRBC membrane was obstructed, suggesting that the actomyosin dependent process could also be involved in parasite egress from the IRBCs. A 10 nM cytochalasin D treatment decreased the merozoites’ motility and the velocity was calculated to be 0.1 µm/sec, while the velocity under 100 nM jasplakinolide treatment was calculated at 0.3 µm/sec. A related study with *T. gondii* tachyzoites showed that a high dose of jasplakinolide inhibited gliding motility, while under some conditions the compound made *T. gondii* tachyzoites more active in gliding [14]. Although *B. bovis* merozoites were also treated with jasplakinolide at 100 µM, only an inhibition of gliding activity was observed at this concentration and not an increase in activity.

**Table 1:** Effects of inhibitors on *B. bovis* motility as determined by time-lapse video microscopy.

	Distance traveled in microns per second
Control	1.2 ± 0.2
Cytochalasin D (1 µM)	−
(10 nM)	0.1 ± 0.1
Jasplakinolide (1 µM)	−
(100 nM)	0.3 ± 0.2

Estimates of rates of motility were based on ten documented individual parasites from two independent experiments. (−) indicates no motility observed with at least three experiments.

### RBC Attachment and Invasion

**Figure 4:**
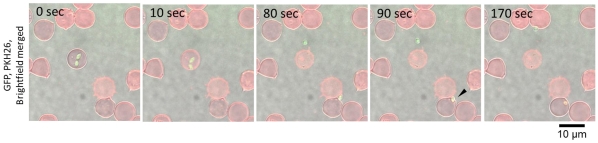
Time-lapse video microscopy of *B. bovis* merozoite egress from a RBC, gliding to and invasion of a new RBC. The arrow head shows merozoite invasion into a new RBC. The red ring-shaped fluorescence around the merozoite indicates the parasitophorous vacuole. The time elapsed between each frame is indicated in seconds.

To observe the invasion of merozoites into new RBCs we recorded a sequence of merozoite motility after the rupture of the IRBC. To enable monitoring of merozoite invasion, cell membranes of bovine RBCs were stained with the red fluorescent dye, PKH26. We observed numerous instances of parasites having successfully invaded fresh RBCs (Fig. 4 and Video S3). Some merozoites attached to several RBCs during their gliding migration before final invasion into a RBC. The ring-shaped red fluorescence around the green merozoite indicated its successful invasion into the new RBC, and it also suggested formation of the PV, which was observed just after the merozoite invasion. It should be noted that when merozoites detached from the RBCs without invasion, the merozoite-RBC interaction was strong enough that pulling of the RBC membrane by the departing merozoite could be observed (Video S4).

### Formation and Breakdown of the Parasitophorous Vacuole (PV)

**Figure 5:**
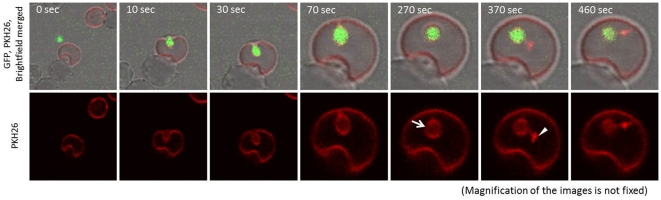
Time-lapse video microscopy of the formation and breakdown of the parasitophorous vacuole. To enable monitoring the PV, PKH26 stained fresh RBC was added to the unstained IRBCs. PKH26 fluorescence can be observed around the merozoite just after invasion into the new RBC (arrow). Subsequently, red dot-shaped fluorescence appears (arrow head), and the ring-shaped fluorescence becomes weaker. The time elapsed between each frame is indicated in seconds.

Next, we monitored the fate of the PV, which was observed as a red fluorescent membrane around the parasite. From the video images, a strongly fluorescent dot appeared beside the parasite and, simultaneously, the ring-shaped red fluorescence disappeared (Fig. 5 and Video S5). The dot fluorescence appeared within 10 min after the invasion of the merozoite into the RBC (the Video S5 shows it appearing approximately 300 sec after invasion). This result suggested that the PV was broken down within several minutes of merozoite invasion into the RBC.

## Discussion

To our knowledge this is the first time that *B. bovis* merozoites have been demonstrated as displaying gliding behavior *in vitro*. Gliding motility of apicomplexan parasites was recorded in time-lapse video images by early work in *Eimeria* sporozoites [15], and detailed observation of such locomotion has been conducted mainly in sporozoites of *Plasmodium* spp. and tachyzoites of *Toxoplasma gondii* (former reports and our findings are summarized in Table 2) [2], [4], [5], [16], [17], [18]. Although both *Plasmodium* and *Babesia* merozoites parasitize host RBCs, *Plasmodium* merozoites observed *in vitro* do not move across a substrate at all, until contacting the RBC surface [19]. From the analysis of the gliding motility, it seems that *Babesia* merozoites can glide in any direction. The merozoites of *B. bovis* rotated their body around the long axis, which was similar to the helical gliding mode of motility defined in *Toxoplasma gondii* tachyzoites, for which the force is generated by the actomyosin motor using myosin A proteins anchored on the inner membrane complex, a specialized membranous structure beneath the parasite's plasma membrane [4]. The rotating direction was counterclockwise, which was also similar to *T. gondii*. In *T. gondii*, it is proposed that closely arranged particle on the inner membrane complex may be connected to the subpellicular microtubule and that the subpellicular microtubule was necessary for the gliding motility [14], [20]. In *Babesia* parasite, the existence of subpellicular microtubule was also reported in *B. major* [21] and freeze-fracture electron microscopy of the intraerythrocytic *B. ovata* merozoites have shown helically arranged intramembranous particles on the inner membrane (S. K., unpublished data), suggesting that there might be helical arrangements of the submembrane cytoskeleton in *Babesia* merozoites, and that the direction of this arrangement is consistent with the counterclockwise rotation of *B. bovis* merozoites. On the other hand, we did not observe other modes of gliding. In contrast to the crescent shape of *Toxoplasma* tachyzoites and *Plasmodium* sporozoites, *B. bovis* merozoites have a pyriform shape. It seems that this pyriform shape provides *B. bovis* merozoites with flexibility in the direction of movement, and therefore the other two forms of motility (circular gliding, upright twirling) observed in *Toxoplasma* tachyzoites might not be necessary. The cell membrane staining of bovine RBCs facilitated confirmation of the successful invasion of merozoites into the RBCs, which was observed by z-stack analysis from confocal laser microscopy. The recorded images revealed that gliding motility led to invasion RBCs by *B. bovis* merozoites by a different manner to the well confirmed process in *Plasmodium* merozoites [22]. In our study, after the attachment of *B. bovis* merozoites, clear reorientation was not observed. Although further detailed analysis is necessary, the video images suggest that the *B. bovis* merozoite might act like a screw as it enters the RBC rather than following the serial invasion process observed for *Plasmodium* merozoites. The force generated by the actomyosin motor during gliding motility is transmitted to the outside of the merozoite through interaction of the motor with a transmembrane protein that serves as an adhesion molecule to the substrate. Recent studies in *Plasmodium* have highlighted the essential role of the thrombospondin related anonymous (or adhesive) protein (TRAP) family in gliding and cell invasion of the parasites [23], [24]. In *Babesia* spp., several TRAP family genes have been identified [25]. However, the role of the TRAP-family in gliding motility of *Babesia* merozoites is still unclear.

**Table 2:** Comparison of gliding motility among apicomplexan parasites.

	*Babesia bovis*	*Plasmodium* spp.	*Toxoplasma gondii*
Gliding stages	Merozoite	Sporozoite, Ookinate[**^2^**]	Sporozoite, Tachizoite[**^2^**]
Gliding Mode	Helical gliding	Stick-and-slip, Circular gliding, Back-and-Forth[**^16^]**, [**^17^**]	Helical gliding, Circular gliding, Twirling[**^4^**]
Gliding Speed	1.2 ± 0.2 µm/sec	1 – 2 µm/sec[**^12^**]	1.1 – 2 µm/sec[**^4^**]
Gliding trails	Can be detected	Can be detected[**^9^**]	Can be detected[**^4^**]
Cytochalasin D	Inhibits gliding, inhibits merozoite egress at high concentration	Inhibits gliding[**^18^**]	Inhibits gliding[**^4^**]
Jasplakinolide	Inhibits gliding	Inhibits gliding[**^18^**]	Inhibits gliding at high concentration, accelerates at low concentration[**^5^**]
Parasitophorous vacuole	Breakdown within 10 min	Remains[**^24]^**, [**^25^**]	Remains[**^4^**]

The experiments with the membrane-stained RBCs enabled us to analyze the PV formation in *B. bovis*. Since neither the IRBCs nor the merozoites were stained with PKH26 in Fig. 5, the appearance of the ring-shaped red fluorescence around the merozoite indicated that the PV originated from the cell membrane of newly infected RBC. Time-lapse video images revealed that a strongly fluorescent dot appeared beside the merozoite within 10 min after the invasion, and that the ring-shaped fluorescence disappeared. This observation supports the idea that the *B. bovis* merozoites rapidly escape or are released from the PV. The video images indicate the timing of the event, whereas the molecular mechanism behind this phenomenon is still unclear. Studies of the blood stage of *Plasmodium* parasites have revealed that the PV membrane is retained until late schizogony [26], [27]. This finding suggests that the PV of *B. bovis* may not be formed in the same way or sealed as securely as in *Plasmodium*. Alternatively, because the PV formation is coupled with the merozoite invasion, the difference in the invasion process by these two parasites may result the difference in the PV formation: in contrast to a relatively slow serial invasion process of *Plasmodium* merozoites, quick vivid invasion of *Babesia* merozoites might be too strong to form PV in the RBC and forced this parasite to survive in RBC without forming PV.

In conclusion, our study of *B. bovis* movement is the first real-time characterization of its motility. How gliding motility functions *in vivo* and regulated is of great interest, especially with respect to future drug and vaccine development. Since gliding motility is not just restricted to *B. bovis* but also found in most members of the apicomplexan group, further study will provide new insight into the molecular mechanisms of gliding motility of these parasites. In addition, we monitored the fate of the PV and revealed the timing of PV breakdown. The escape or release of the merozoite from the PV is a unique point in the lifecycle of *Babesia* parasites, and it seems to be a critical process for the parasite to establish the intraerythrocytic growth stage. Comparative genome analysis between *Babesia* and *Plasmodium* in terms of PV formation might be an interesting subject to be addressed in future studies.

## Materials and Methods

### Ethics Statement

Bovine RBCs were collected every two weeks from healthy animals. This study was performed in strict accordance with the recommendations in the Guide for the Care and Use of Laboratory Animals of the Obihiro University of Agriculture and Veterinary Medicine. The protocol was approved by the Committee on the Ethics of Animal Experiments of the Obihiro University of Agriculture and Veterinary Medicine (Permit number 23–26).

### Parasite Culture

The *Babesia bovis* Texas strain was maintained in purified bovine RBCs with GIT medium (WAKO, Osaka, Japan) by a microaerophilic stationary-phase culture system [28]. The parasites were cultured in 1 ml culture medium containing 10% bovine RBCs in 24-well culture plates (Corning, NY, USA). Culture medium was replaced every day and the level of parasitemia was monitored daily by staining thin blood smears with Giemsa solution.

GFP-fluorescent *B. bovis* populations were established previously [13]. Briefly, the GFP-expressing plasmid was constructed. Then, the DHFR expression cassette was cloned into the plasmid with the GFP expression cassette. The plasmid constructs were introduced into the Texas strain of *B. bovis* by transfection with a Nucleofector® device (Amaxa Biosystems, Cologne, Germany). The transfected parasite population was selected with 5–10 nM of WR99210 and the parasite population with GFP expression was cloned using a limiting dilution. The fluorescent parasite was maintained for more than 7 months under WR99210 drug pressure.

### Video Microscopy

*In vitro* cultured IRBCs were applied to glass slides for time-lapse analysis. Time-lapse video microscopy was conducted using a TCS-SP5 confocal laser scanning microscope (Leica Microsystems, Wetzlar, Germany). Confocal fluorescence images and transmitted images were recorded digitally at approximately one frame per second (0.8–1.5 frame/sec). Time-lapse images were recorded continuously for up to 30 min at room temperature (RT) and merozoite egress from the IRBC and their gliding were monitored. Frames used to create the time lapse series were taken from avi movies and processed using AviUtl software.

### Immunofluorescence Detection of Trails

Staining of surface protein in trails was performed by indirect immunofluorescence using the antiserum against *B. bovis* merozoite surface antigen 1 (BbMSA-1) [29]. Glass slides were coated in 10 µg/ml poly-L-lysine in PBS for 30 min at RT and rinsed in phosphate buffer saline (PBS). IRBCs were resuspended in GIT medium, added onto the slides, and incubated at 37°C for 30 min. Slides were briefly washed by PBS, dried and fixed with 50% acetone-50% methanol for 5 min at -20°C. Anti-BbMSA-1 mouse antiserum was used at 1∶50 and Alexa-Fluor 488 conjugated goat anti-mouse IgG (Molecular probes, OR, USA) was used at 1∶200. The slides were incubated with each antibody for 45 min at RT, and then observed with a TCS-SP5 confocal laser scanning microscope.

### Gliding Inhibition Assays

For the gliding inhibition assays, cytochalasin D (Wako Pure Chemical, Osaka, Japan) and jasplakinolide (Enzo life sciences, NY, USA) was dissolved in DMSO at 1 mM and stored at −30°C. IRBCs were resuspended in GIT medium, and treated with 10 nM or 1 µM cytochalasin D, 100 nM or 1 µM of jasplakinolide, or 0.1% DMSO for 30 min, respectively. Parasite tracks were traced from the pictures onto transparent sheets and gliding velocity was analyzed for each merozoite. Velocities were calculated over periods of five seconds and the highest speed was taken as the gliding speed. A total of 10 merozoites from two independent experiments were analyzed to obtain mean ± SD values.

### Mitochondrial Staining of *B. bovis*

To stain the mitochondria of *B. bovis* merozoites, MitoTracker Red CM-H_2_XRos (Molecular Probes, OR, USA) was used. The fluorescent probe was diluted to 200 nM in GIT medium, and mixed with an equal amount of IRBCs. The mixture was incubated for 30 min at 37°C and washed with GIT medium 3 times. Parasites with mitochondrial staining were used for analysis on mode of gliding.

### Membrane Staining of the Bovine RBCs

To stain the cell membrane of the bovine RBCs, a PKH26 red fluorescent cell membrane labeling kit was used (Sigma-aldrich, MO, USA). Fifty micro liters of packed bovine RBCs were washed once with PBS, then 250 µl of diluent C followed by 1 µl of PKH dye stock were added to the RBCs. The reaction mix was incubated for 10 min at RT. After staining, RBCs were washed 3 times with PBS and used for time-lapse image analysis.

## Supporting Information

Video S1 **Time-lapse video microscopy of a** ***B. bovis*** **merozoite engaged in gliding.** Video S1 is shown at 6.7× real time.
(AVI)

Video S2 **Helical motility of** ***B. bovis*** **merozoites.** Video S2 is shown at 2.8× real time.
(AVI)

Video S3 **Time-lapse video microscopy of** ***B. bovis*** **merozoite egress from a RBC, gliding to and invasion of a new RBC.** Video S3 is shown at 15.4× real time.
(AVI)

Video S4 **Attachment of** ***B. bovis*** **merozoites on the RBC.** Furrowed RBC surface could be observed at the position where merozoite attached. The video is shown at 15.4× real time.
(AVI)

Video S5 **Time-lapse video microscopy of the formation and breakdown of the parasitophorous vacuole.** Video S5 is shown at 15.4× real time.
(AVI)
